# Supplementary material for: A Possible Preventive Role of Physically Active Lifestyle during the SARS-CoV-2 Pandemic; Might Regular Cold-Water Swimming and Exercise Reduce the Symptom Severity of COVID-19?
Source: Int J Environ Res Public Health. 2021 Jul 4;18(13):7158. doi: 10.3390/ijerph18137158 (PMC8297290; doi:10.3390/ijerph18137158)
Supplement: Supplementary file 1 [file ijerph-18-07158-s001.zip › ijerph-1227772-SI.pdf]

Table S1. Risk ratios for upper resiratory tract infection by gender.

**Upper resiratory tract infection (frequency/year)**	Model 1 male		Model 1 female		Model 2 male		Model 2 female	
**<1 time**	0.837 [0.662, 1.058]	0.07	1.1894 [0.931, 1.519]	0.08	1.562 [1.267, 1.927]	0.00	2.172 [1.705, 2.767]	0.00
1 time	1.229 [0.935, 1.616]	0.07	1.094 [0.873, 1.370]	0.22	0.756 [0.548, 1.042]	0.04	0.591 [0.400, 0.873]	0.01
2 times	1.532 [0.922, 2.547]	0.05	0.708 [0.528, 0.949]	0.01	0.535 [0.279, 1.026]	0.03	0.474 [0.290, 0.775]	0.01
>2 times	0.500 [0.282, 0.889]	0.01	0.938 [0.579, 1.518]	0.40	0.168 [0.068 , 0.413]	0.00	0.432 [0.181, 1.029]	0.03

Model 1 physical activity. Model 2 physical activity + winter swimming
